# From intuition to AI: evolution of small molecule representations in drug discovery

**DOI:** 10.1093/bib/bbad422

**Published:** 2023-11-29

**Authors:** Miles McGibbon, Steven Shave, Jie Dong, Yumiao Gao, Douglas R Houston, Jiancong Xie, Yuedong Yang, Philippe Schwaller, Vincent Blay

**Affiliations:** Institute of Quantitative Biology, Biochemistry and Biotechnology, University of Edinburgh, Edinburgh, Scotland EH9 3BF, United Kingdom; Institute of Quantitative Biology, Biochemistry and Biotechnology, University of Edinburgh, Edinburgh, Scotland EH9 3BF, United Kingdom; Xiangya School of Pharmaceutical Sciences, Central South University, Changsha, 410013, China; Institute of Quantitative Biology, Biochemistry and Biotechnology, University of Edinburgh, Edinburgh, Scotland EH9 3BF, United Kingdom; Institute of Quantitative Biology, Biochemistry and Biotechnology, University of Edinburgh, Edinburgh, Scotland EH9 3BF, United Kingdom; Key Laboratory of Machine Intelligence and Advanced Computing, Sun Yat-Sen University, Guangzhou, 510000, China; Key Laboratory of Machine Intelligence and Advanced Computing, Sun Yat-Sen University, Guangzhou, 510000, China; Laboratory of Artificial Chemical Intelligence (LIAC), Institut des Sciences et Ingénierie Chimiques, Ecole Polytechnique Fédérale de Lausanne (EPFL), Lausanne, Switzerland; Institute of Quantitative Biology, Biochemistry and Biotechnology, University of Edinburgh, Edinburgh, Scotland EH9 3BF, United Kingdom

**Keywords:** drug discovery, autoencoders, transformers, artificial intelligence, SMILES, machine learning

## Abstract

Within drug discovery, the goal of AI scientists and cheminformaticians is to help identify molecular starting points that will develop into safe and efficacious drugs while reducing costs, time and failure rates. To achieve this goal, it is crucial to represent molecules in a digital format that makes them machine-readable and facilitates the accurate prediction of properties that drive decision-making. Over the years, molecular representations have evolved from intuitive and human-readable formats to bespoke numerical descriptors and fingerprints, and now to learned representations that capture patterns and salient features across vast chemical spaces. Among these, sequence-based and graph-based representations of small molecules have become highly popular. However, each approach has strengths and weaknesses across dimensions such as generality, computational cost, inversibility for generative applications and interpretability, which can be critical in informing practitioners’ decisions. As the drug discovery landscape evolves, opportunities for innovation continue to emerge. These include the creation of molecular representations for high-value, low-data regimes, the distillation of broader biological and chemical knowledge into novel learned representations and the modeling of up-and-coming therapeutic modalities.

## DIGITAL FORMATS FOR SMALL MOLECULES

Describing molecules has taken many forms throughout history and disciplines. Initially, uniquely naming molecules with ‘common names’ was sufficient. Understanding the atomic makeup of molecules enabled a systematic designation of common moieties and their positions on molecular graphs. In 1919, the International Union of Pure and Applied Chemistry (IUPAC) was formed to standardize chemical nomenclature and terminology, devising a ‘preferred IUPAC name’ for molecules [1]. While this nomenclature permits a thorough description of molecules, it can be difficult to understand and requires an encyclopedic knowledge of chemical moieties, naming conventions and syntax. Alternative formats better suited for computers [2] soon appeared to compactly and accurately capture compounds of different sizes with specializations for small molecules, macromolecules, polymers and crystals. Currently, the choice of format is vast, with the open-source Open Babel program supporting 146 different molecular formats. Formats fall broadly into two categories: molecular-graph-based formats and 3D molecular formats.

A popular molecular-graph-based format is Simplified Molecular-Input Line-Entry System (SMILES) [3], which represents molecules as a string of characters. For example, the popular drug acetaminophen can be represented in SMILES format as ‘CC(=O)Nc1ccc(O)cc1’. This format has the advantage of being easily human- (with practice) and machine-readable. However, there are multiple synonymous ways to write the same molecule using this format. One can define rules to select the ‘canonical’ SMILES representation of a molecule, but different toolkits often have different rulesets, which can complicate comparisons. Extensions and alternatives to the SMILES format were developed, including SMARTS, SMIRKS, OpenSMILES, SYBYL Line Notation [4] and, recently, SELFIES [5]. IUPAC also created the InChI format [6]. This hierarchical string format is difficult for humans to interpret, but easily machine-readable, comprising distinct layers of information, including charge, stereochemistry and isotope information.

Molecular-graph-based formats do not typically include specific spatial information of the atoms besides connectivity. 3D molecular formats [7], such as MOL and MOL2, describe atoms and their positions in Euclidean space, often accompanied by additional information such as partial charge. Wigh *et al.* [8] provide a comprehensive breakdown of the MOL and related Chemical Table (CTAB) formats. While the MOL and MOL2 formats are comparable in terms of size, metadata and human readability, MOL is more widespread, often with multiple molecules in MOL format concatenated into a single Structure Data File (SDF) file. SDF is a very popular approach for representing small molecules with 3D information outside of crystallography, molecular dynamics and other specialized disciplines.

As the field of computational drug discovery matured, various ways to feed molecules into mathematical models were explored (Table 1). Often these approaches only leverage molecular connectivity information, since structural and dynamic information can be expensive to measure or compute and increases storage requirements. In the early days of the cheminformatics and QSAR fields, hand-crafted designer encodings were invented, such as molecular descriptors and fingerprints (section Bespoke Representations). With the advent and rise of deep learning since the 2010s, these approaches have been complemented by learned representations that can directly ingest strings (e.g. SMILES) or molecular graphs (section Learned Representations). Currently, SMILES and graph encodings have become the standard representation of small molecules for use in neural network models. But this is not the end of the story: there is ample room for improvement, and conventional small molecules are only a portion of the growing chemical space of pharmaceutical interest (section Conclusions).

**Table 1:** Comparison of different formats for digital representation of molecules

Qualities captured	Examples	Encoding efficiency	Human-readable?
**Connectivity** The most compact and simple way to represent a molecule with atom/group connectivity information.	IUPAC name, SMILES, SMARTS, InChI	Low storage requirements. Low encoding efficiency. Little information captured besides connectivity.	Readable.
**Atom connectivity and 3D conformation** The most common 3D small molecule file storage formats. Requiring potentially costly conformation generation steps to move from connectivity representations to 3D molecules.	MOL, MOL2, PDB, SDF, CTAB	High storage requirements, potentially suboptimal ASCII text in a structured format containing repeats; may be compressed.	Not easily read. Conversion to connectivity formats for visualization is preferred.
**Molecular descriptors** Basic computable properties of a molecule, like the MW, number of hydrogen bond acceptors/donors, etc. Useful in filtering and applying rulesets.	MW, logP, 3D descriptors like USRCAT [9] and FEPOPS [10]	Low storage requirements, although often suboptimal because of correlated descriptors.	Yes; particularly useful to drive Med Chem decision-making, with their inclusion in many rules of thumb, like Lipinski’s Ro5 [11] and QED [12].
**Fingerprints** Powerful molecular representations of molecules traditionally used in QSAR studies and early AI/ML cheminformatics [13]. The popular ECFP4 fingerprint was used extensively in QSAR studies	FP4, MACCS [14], Morgan, ECFP [15], FCFP, MinHashed [16]	Low storage requirements; efficient encoding historically used for catalog searching using fingerprint lookups.	No; while decoding of bit information may be possible indicating feature presence (not connectivity), bit strings are often folded to a certain length and then encoded to integers.
**Learned representations** The broadest family, typically using real numbers to describe a molecule and its predicted properties.	Graph-based and latent space embeddings	Low to medium storage requirements. Often correlated features inflate storage requirements.	No; used to encode molecules in often abstract ways for comparison using tuned distance/similarity functions.

## BESPOKE REPRESENTATIONS

Molecular descriptors are numerical representations of small molecules computed using predefined rules and enable mathematical modeling. The development of molecular descriptors has gone through a relatively long evolution [17–19]. Since the 1960s, descriptors have been proposed to extract quantitative relationships between chemical structure and properties (QSAR), starting with intuitive physicochemical descriptors such as molecular weight (MW) and logP, and topological descriptors [17] that successfully contributed to ubiquitous medicinal chemistry rulesets like Lipinkski’s Rule of 5 for predicting oral availability [20] and other extended rulesets targeting prediction of lead-likeness [21]. Later on, researchers devised more complex E-state electrical descriptors and topological autocorrelation descriptors [22, 23], which capture the underlying structure of the molecule along with physicochemical properties. As computer technology developed, more molecular descriptors were proposed, such as atom-pair descriptors and computationally demanding molecular electrostatic potentials [24–27], which aim to capture fields surrounding molecules that would be experienced or interacted with by another molecule upon binding [27]. By 2000, thousands of molecular descriptors had been proposed, as summarized by Todeschini and Consonni [19].

The calculation of molecular descriptors mainly relies on specialized software packages (Table 2). These generally emerged from one of three popular programming languages: C++, Java and R. RDKit, a C++-based package with an extensively used and ever popular Python interface, provides numerous molecular operations and can currently calculate 208 descriptors and 5 fingerprints. Many cheminformatic packages build on RDKit, including Chemopy [28], PyDPI [29] and PyBioMed [30]. Others extend existing functionality to generate new descriptors such as MACAW [31], whereas others, like Mordred, generate popular descriptors *en masse*. In 2003, Chemistry Development Kit (CDK) [32] was developed in Java, which currently allows calculation of 275 common molecular descriptors and 9 fingerprints. BlueDesc, jCompoundMapper [33] and MOLD2 [34] were later developed, leveraging CDK as the underlying molecular engine for descriptor generation, along with PaDEL [35], which benefits from a user-friendly interface that popularized its use. Built in the R programming language; Rcpi [36] was developed to model relationships between compounds and proteins and allows generating more than 300 small-molecule descriptors. Based on Rcpi, BioMedR was extended to describe nucleic acids [37]. Commercial software such as Alvascience alvaDesc, CCG MOE, BIOVIA Discovery Studio, Schrodinger and SYBYL also provide descriptor generation capabilities, whereas software like ADMEWorks ModelBuilder and PreADMET allow generating descriptors as well as building QSAR models. Besides standalone software, descriptors can also be obtained through webservers such as E-Dragon, ChemDes [38] and BioTriangle [39], or databases with precomputed descriptors, such as PubChem, ChEMBL and DrugBank.

**Table 2:** Examples of common software and webservers used for computing molecular descriptors. ‘Year’ means the date of first release

Name	Year	No. of descriptors	No. of fingerprints	Link	Ref.
Dragon	1997	5270 (v.7.0)	–	http://www.talete.mi.it/	
CDK	2003	275	9	https://cdk.github.io/	[32]
BlueDesc	2003	174	–	http://www.ra.cs.uni-tuebingen.de/software/bluedesc/	
Mold2	2008	779	–	https://www.fda.gov/science-research/bioinformatics-tools/mold2	[34]
Pybel	2008	24	4	https://github.com/pybel/pybel	[57]
PaDEL	2011	1875	12	https://github.com/ecrl/padelpy	[35]
RDKit	2013	196	8	https://www.rdkit.org/	
PyDPI	2013	615	7	https://pypi.org/project/pydpi/	[29]
Chemopy	2013	1135	7	https://github.com/ifyoungnet/Chemopy	[28]
Rcpi	2015	308	10	http://bioconductor.org/packages/release/bioc/html/Rcpi.html	[36]
Mordred	2018	1825	–	https://github.com/mordred-descriptor/mordred	[58]
PyBioMed	2018	775 (9920 protein descriptors, 6000 DNA/RNA descriptors)	19	https://github.com/gadsbyfly/PyBioMed	[30]
alvaDesc	2019	5666 (v.2.0.16)	3	https://www.alvascience.com/alvadesc/	[59]
BioMedR	2021	293 (9920 protein descriptors, 6000 DNA/RNA descriptors)	13	https://github.com/wind22zhu/BioMedR	[37]
E-dragon	2005	1600	–	http://www.vcclab.org/lab/edragon/	
ChemDes	2015	3679	59	http://www.scbdd.com/chemdes	[38]
BioTriangle	2016	540 (9890 protein descriptors, 6376 DNA/RNA descriptors)	7	http://biotriangle.scbdd.com	[39]
ChemSAR	2017	783	10	http://chemsar.scbdd.com	[60]

In practice, each tool has its own advantages and disadvantages. Commercial software offers high stability but requires the purchase of a license. While many standalone packages are highly flexible and can be integrated into drug discovery pipelines, they rely on specific runtime environments and often require programming knowledge. The use of webservers does not require programming knowledge, which expands their user base. However, their calculation processes are often a black box and throughput is limited by the network and server capabilities. While convenient to use in industrial production environments, cutting edge science will benefit from complete control over processes, which we see in the growing adoption of open source software and FAIR principles [40, 41].

Over time, descriptors were increasingly used for quantitative relationship studies on various compounds and their interactions with target proteins [42, 43]. They are also commonly used to calculate and predict basic small molecule properties including ADMET and synthetic accessibility [44] profiles, serving to drastically reduce in silico search spaces and the number of compounds that need to be synthesized. Arguably, their most powerful use is enabled by the ‘molecular similarity principle’ [45], which states that similar molecules should make similar interactions, allowing the retrieval of potential actives based on known actives and enabling further hit expansion, SAR exploration and scaffold hopping [46–48] in order to replace core scaffolds, access new chemistries, change pharmacokinetics, toxicity profiles and chemical space. Further uses include database retrieval, QSAR, molecular docking and structure/pharmacophore visualization for rational drug design [46–49]. Since the advent of machine learning, descriptors have been used as a method of molecular encoding or ‘featurization’ and still offer state-of-the-art performance in many applications as is evidenced in the literature and AI/ML competitions with teams working to score highly in benchmarks such as MoleculeNet [50].

Fingerprints can be considered a subtype of descriptors. They initially represented molecules using bitstrings based on features present within molecules. The distinction is blurry; however, as many descriptors use binary elements to describe a molecule mixed with more complex data types (e.g. FEPOPS [10]). Compared with other molecular descriptors, fingerprints have some desirable characteristics: (i) they are computed and scored/compared using fast and very robust algorithms, (ii) they encode molecules into fixed-sized, information-rich vectors and (iii) powerful fingerprint generators are freely available in software like RDKit. With thousands of descriptors available, feature cleaning and selection can be tedious, making fingerprints a good plug-and-play option to expedite modeling.

Interestingly, quantum machine learning (QML) models are gaining popularity in predicting chemical properties because of their increasing speed and the possibility of generating rich data from first principles to describe molecules in models [51, 52]. Property-based QML models such as AlphaQ [53] used the RHF/6-31G** level of theory to calculate 3D distribution of electrostatic potential in a molecule and used this to train a highly performing neural in the prediction of blood–brain barrier permeability. As an example of a wave-function-based QM model, Schütt *et al.* [54] used atom types and positions as input to a deep neural network to construct representations of the chemical environments of >100 atoms, from which molecular properties such as HF energies, charge populations, bond orders and dipole moments could be obtained. Both property-based and wave-function-based QML models require QM calculation of the input chemical structures, thus, their speed is mainly determined by the computation cost of the level of QM theory incorporated in models. Advances in quantum computing could popularize the application of these and other approaches [55]. In contrast, delta-QML approaches such as DelFTa [56] use 3D message-passing neural networks to replace expensive calculations in density functional theory (DFT) by computationally inexpensive GFN2-xTB approximations, achieving very high speed in estimating molecular properties with DFT accuracy. We believe they could open novel possibilities for molecular representation and property modeling in drug discovery, building more accurate models for the early attempts at capturing electrostatics and field information to encode molecules.

## LEARNED REPRESENTATIONS

The application of machine learning methods to problems in drug discovery is exploding, with impacts seen across structure-based virtual screening, molecular property prediction and molecular similarity. As with bespoke descriptors, the aim of many deep learning approaches to molecular representation is to map a complex molecule into an appropriate embedding rich in useful information for a given task. Unlike molecular descriptors or fingerprints, learned embeddings are potentially lossless and able to extract any information present in the parent molecule, rather than a set of predefined features [61]. Hence, these learned embeddings are arguably more suited to inference of desired properties in downstream prediction tasks compared with traditional descriptors or fingerprints. While many deep learning models applied to small molecules are trained to predict regression values or classification labels, they produce learned embeddings internally from which these predictions are inferred. It is these learned embeddings that are the subject of this section.

Table 3 summarizes some architectures discussed in the following subsections. Different algorithms have different inductive biases, which refer to the family of functions that the algorithms can entertain. The shape or structure of this hypothesis space (i.e. which functions are in it, which are not and which are deemed more probable or preferable) provides the algorithm’s inductive bias. The skill in machine learning often lies in matching the problem with an algorithm (or family of functions) that has a helpful inductive bias. While the best inductive bias depends on the specific problem, graph- and string-based encodings, in combination with expressive graph-neural networks and transformer architectures, have become extremely popular. However, they often require more data to train and generalize than models with a more restrictive hypothesis space. To alleviate this, we will see that pretrained embedders can be used for any encoding approach. They leverage unsupervised pretraining on large unlabeled molecular data sets to learn generalizable representations before fine-tuning on downstream tasks. This pretraining helps mitigate overfitting, especially when labeled data is scarce, by exposing the model to much more diverse molecules than typically seen during supervised training.

**Table 3:** General characteristics of some common machine learning approaches used to generate molecular representations

Approach	Input representations	Inductive bias	Usage
Convolutional encodings	1D strings, 2D images, 3D grids	Translation invariance through weight sharing	Use CNNs to extract features from grid-based inputs
Graph encodings	Molecular graphs—atoms as nodes, bonds as edges	Rotation invariance through graph structure	Use graph neural networks to aggregate atom neighbor features
String encodings	SMILES strings, other sequential formats	Context through sequential position encoding (and self-attention)	Use RNNs, transformers and autoencoders on sequence

### Convolutional encodings

Convolutional neural networks (CNNs) came to force for their utility in image recognition tasks, with LeNet being one of the first high-performance architectures [62]. CNNs have been similarly applied to images of small molecules. A convolution in this context refers to a mathematical operation by which a compact tensor of learnable values (a so-called filter) is multiplied and summed over the larger input tensor. This strategy allows the model to learn to extract higher-level features from anywhere in the input in a very efficient way. The strength of CNNs stems from these convolutions, and an input data tensor is transformed by the result of multiple convolutions performed on each image or sample, resulting in a weighted representation of aggregated local patterns. In LeNet [62] and many following models, this weighted representation of local patterns is pooled and fed to a dense linear layer to perform a classification or regression task. This internal representation is also a form of learned embedding.

Convolutional embeddings have been extensively applied to molecular representations, starting from different molecular formats. For instance, Chen *et al.* [63] used one-hot encoding of SMILES strings as matrices, with each matrix column containing binary encodings of atom-specific chemical features. Uesawa [64] developed a QSAR predictive model trained on batches of images of small molecules. Hirohara *et al.* [65] applied CNNs to one-hot encoded matrices of small molecules. Yuan *et al.* [66] applied a CNN to a 0.5 × 0.5 Å resolution 2D grid representation of molecules detailing the van der Waals forces present in each cell. Voxel grids representing the electron density of a specific molecule’s conformation in 3D space have also been used as inputs to CNNs: Kuzminykh *et al.* [67] used a voxel-based encoding of 3D small molecules as inputs to a convolutional autoencoder. Wang *et al.* used CNNs with several 3D point cloud representations of protein-ligand systems, where ligand and protein atoms from complex structures were encoded by their cartesian coordinates, atom identities and a binary feature indicating protein or ligand. The approach achieved accurate binding affinity prediction on a CASF-2016 benchmark set [68].

Nevertheless, CNN-based embeddings have limitations, such as the lack of rotation-invariance. The embedding produced from a given input image or 3D electron density grid may change if the molecule is rotated [69], which can lead to different predictions for a given molecule. The same issue is true of one-hot encodings of equivalent non-canonical SMILES strings. Many works circumvent this issue by augmenting the input data with many rotations and translations of the same training molecule or enumeration of valid SMILES strings representing the molecule, aiming to force the network to identify the molecular signal within the data and become invariant to the rotational representational noise. Augmentation of input representations in this manner is critical for the models to generalize but hugely increases the training time and computational cost. This limitation of the CNN architecture led to the favored adoption of graph encodings for molecular representation. A promising research direction is equivariant neural networks, which incorporate Euclidean symmetry into the model and overcome the need for data augmentation [70].

### Graph encodings

In geometric (or graph-based) deep learning, atoms are represented as nodes in a graph, with bonds between atoms represented by edges [71]. Nodes and edges have associated features: node features typically detail atom characteristics (e.g. chemical element and aromaticity) and edge features indicate bond types (e.g. single, double and triple) (Table 4). In this way, a whole molecule is represented as a single graph. Since graphs do not typically contain data on the absolute positions of atoms, graph neural networks allow near-perfect, rotation-invariant representations. Graph representations are well suited to deep learning methods and can lead to superior performance over designer representations like fingerprints [72]. Geometric deep learning has been extensively applied to molecular property prediction [71], including solubility, toxicity and oral bioavailability [50, 73, 74].

**Table 4:** List of commonly used node and edge features when encoding small molecules as molecular graphs

Node features	Edge features	Edge connectivity
-One-hot encoded atom identity -Atomic number -Partial charge -Aromaticity -Hybridization -Hydrogen bond donor/acceptor status -Number of bonded neighbors (including or excluding hydrogen atoms) -Explicit valence -Implicit valence -Formal charge -Number of radical electrons -Chirality -Mass	-One-hot encoded bond type (single, double, triple, aromatic) -Float bond length in angstroms -Conjugation status -Float bond angle -Stereo configuration	-Undirected edges drawn between atoms with chemical bonds -Directed edges drawn between atoms with chemical bonds -Fully connected (all atoms to all atoms) -Each atom connected to k-nearest atoms

**Figure 1:**
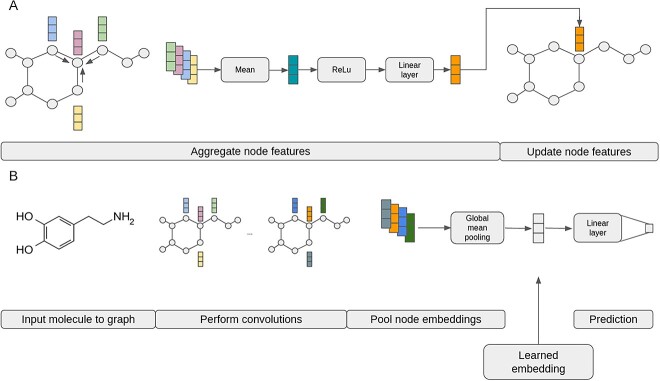
Illustration of convolution and pooling over a small molecule graph as part of a neural network model. (**A**) Detailed schematic of node feature updating by a single convolution performed on a single node. (**B**) High level view of whole graph embedding involving a molecular graph, multiple convolutional layers, global pooling and a linear layer for predictions.

Molecular graph classification and regression tasks are commonly achieved using multiple convolutions in a graph convolutional network [75]. During a convolution, for each node in the molecular graph, the node features of neighboring nodes (connected by edges) are aggregated with each node’s own features using a given function (e.g. the mean or the sum of the given node’s features) (Figure 1A). This aggregated node representation is then passed through a simple dense layer with a non-linear activation function to produce a weighted aggregated representation of arbitrary size *J* for each node (Figure 1A) [75]. Note that the weights of these simple layers are trainable parameters. Each nodes’ features are then updated with their new weighted aggregated forms (Figure 1A). Applying multiple sequential convolutional layers adds context of nodes further away as the aggregations travel through the graph [75]. Thus, few convolutional layers restrict features richer in local information, whereas many convolutional layers produce node features containing more global information from more nodes in the graph.

To obtain a 1D vector representation of the whole graph with fixed size, a form of pooling is employed to aggregate the node representations (Figure 1B). For example, for an input graph with 10 nodes and a hidden dimension *J* of size 64, each of the node’s updated features would be concatenated to a matrix of size 64 × 10. This matrix could be pooled horizontally using a sum or mean function, giving a final representation of size 64 representative of all nodes in the graph [76]. These pooling methods, therefore, result in a fixed-size vector *J* for any given number of nodes (Figure 1B). The resultant pooled embeddings of small molecules produced by geometric deep learning methods contain both local information (e.g. the presence of a hydroxyl group) and global information (the location of that hydroxyl group relative to other groups) [77]. In supervised learning, training data often consist of pairs of small molecules and their respective ground truth labels for a classification task (e.g. drug-likeness) or a regression task (e.g. logP prediction). In such cases, this embedding is generally hidden within the network and is connected to a final linear layer to predict the label associated with the input graph (Figure 1B). Although much less common, some examples of contrastive learning to generate molecular embeddings have also been reported [78, 79].

### String encodings

String encoding of small molecules involves two steps. Initially, string representations of small molecules are converted to a numerical representation of the string characters. Frequently, these are integer tokens (e.g. C:1, =:2, O:3) or 2D binary one-hot encoding matrices (where a 1 in a column represents a SMILES symbol, and a 1 in a given row represents the position of the character in the SMILES string).

The ability to use SMILES strings to represent small molecules in deep neural networks through techniques like one-hot encoding was arguably a turning point, perfectly capturing the molecular graph of a molecule. This was demonstrated to great effect by Gómez-Bombarelli *et al*. [80] who used an autoencoder deep neural network architecture (see below) to embed small molecule SMILES in a numerical space (latent space). Of key importance was the functionality to reconstruct SMILES from points within this latent space, creating a generative method operating on a continuous chemical space. While representing molecules this way was undoubtedly less noisy and efficient than operating on images of molecules, it was not without issues in that many areas of the latent space decoded to invalid SMILES strings containing unclosed brackets and incorrect bond orders. These issues were particularly visible when interpolating between molecules within the latent space, or exploring areas not well represented in training data. In an attempt to overcome this, additional line-based representations were developed such as DeepSMILES [81] and SELFIES [5], which aimed to alleviate the need of learning the details of SMILES syntax to generate valid strings and provide a robust route to mapping to small molecules. Wigh *et al*. [8] provided an in-depth review of the autoencoder architecture as applied to encoding small molecules into a conditioned embedding space. Furthermore, models trained on encoded strings are limited to their training set encoding vocabulary; molecules containing elements not seen during training have no possible encoding equivalent. Hence, these molecules cannot be adequately encoded, which can limit the generality of models trained on small data sets in this manner.

While convolutional approaches and autoencoders have been popular in encoding one-hot matrices of string representations, more recently, the transformer architecture has shown state-of-the-art performance in string encoding and decoding tasks [82], gaining popularity for modeling many types of sequence data in biology, such as small molecule strings, protein sequences and DNA sequences [83–85].

### Pretrained embedders

The above-discussed architectures and subsequent learned embeddings can be useful for supervised training on molecular prediction tasks [86]. However, the combination of a small data sets and complex models with many parameters often results in failure to find truly good, optimized model parameters [87]. Hence, the learned embeddings of small molecules produced within these models can lack key information about the input molecule, be overly specific to the training data, focusing on specific areas of chemical space explored in training data as a function of the chosen loss function and subsequently fail to generalize to unseen samples absent from training. For this reason, the production and use of pretrained embedders for molecular representation have increased rapidly in recent years. The aim of these embedders is not to perform well on a single given property prediction task, but rather solely to produce a rich initial embedding of a molecule in latent space [88] and fully capture a lossless representation of the molecule. To this end, models are commonly trained in an unsupervised manner [89]. Unsupervised training involves unlabeled data; molecules without any specific property or endpoint being considered. These models then have much more data available to them and if trained carefully, are more likely to converge on good parameters, enabling them to produce powerful, generalizable embeddings of new input molecules [89].

Pretrained embedders are commonly trained by being asked to encode a given input molecule in latent space, and subsequently decode the latent space embedding back to the ground truth input given some challenging condition, such as an information bottleneck as is the case for the autoencoder architecture, or a partial (masked) input. While in principle any network architecture could be trained in an unsupervised manner, the most common architectures for unsupervised learning of molecular representations are autoencoders and transformers. Irwin *et al.* [90] applied the partial input masking technique in training Chemformer, using a randomly sampled subset of 100 million molecules from ZINC 15, and achieved state-of-the-art performance in synthetic benchmarks. Zeng *et al.* [91] employed an extensive repertoire of pretraining methods to produce a single high-performing molecular encoder for the virtual screening of anti-SARS-CoV-2 molecules, including molecular image reconstruction and masked contrastive learning.

**Figure 2:**
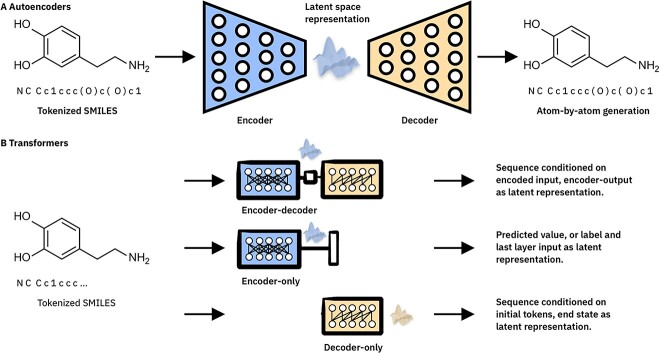
High-level comparison of machine learning architectures used for unsupervised learning of molecular representations. (**A**) Autoencoder architectures consist of encoder, decoder and bottleneck layer elements with the latter being the location of the learned embedding representation. (**B**) Transformer architectures, which all consist of preprocessing and positional embedding steps, followed by multiple sequential encoder and/or decoder blocks. Top: Sequence-to-sequence original or BART-style Transformer, where the encoder output can be aggregated as learned embedding representation. Middle: Encoder-only BERT-style Transformer, typically fine-tuned on molecular classification/regression. The output of the encoder module can be used as a learned embedding representation. Bottom: Decoder-only GPT-style Transformer, where the end state can be used as a learned embedding representation.

The autoencoder architecture has two main elements: the encoder and the decoder. SMILES string encodings are passed through a series of increasingly small encoder layers to the bottleneck layer. The decoder module then attempts to take the bottleneck layer output representation and reconstruct the original input (Figure 2A). Hence, the model must learn to efficiently represent the entire molecule as a small, fixed-size vector to minimize the loss between the inputs and the reconstructed outputs. Traditionally these models consist of standard dense linear layers which decrease and increase in size sequentially, although graph autoencoders have also been employed in this field [92]. This autoencoder-based approach was employed by Gómez-Bombarelli *et al*. [80] to produce rich latent space embeddings of small molecules that they leveraged for generative purposes.

Since the release of the transformer architecture in 2017, transformers rapidly gained popularity [93]. Large language models (LLMs) based on the transformer architecture are receiving much attention because of unprecedented performance of Generative Pretrained Transformer (GPT) language models in natural language tasks [94]. In the same way, Bidirectional Encoder Representations from Transformers (BERT) and Bidirectional Autoregressive Transformer (BART) style models have been extensively applied to learn embeddings of string representations of small molecules (Table 5). Transformers consist either of encoder-only (e.g. BERT), decoder only (e.g. GPT) or of encoder and decoder modules trained simultaneously (Figure 2C)^79^. Alternatively, BERT-style transformers train multiple sequential encoder blocks without decoder blocks, using a simple classifier layer to return embeddings to string inputs. While autoencoders employ a bottleneck layer between encoding and decoding, bidirectional transformer training for molecular representation is commonly achieved with a process called masking. String representations of molecules are initially tokenized. These tokenized representations have a subset of their elements randomly hidden; this is the masking process. Finally, the transformer is shown masked tokenized SMILES strings as input data and asked to predict the unmasked original string as an output (Figure 2B). To perform well at predicting the masked characters, the transformer must learn the general ruleset of valid strings. Hence, once trained the encoder module produces powerful, chemically informed embeddings of input molecules. Small molecule encoders, such as Chemformer, have shared in the outstanding results of transformer-based sequence models (Table 5) [90].

**Table 5:** List of popular small molecule pretrained embedders

Name	Year	Training data	Architecture	# of parameters	Link	Ref.
Chemical VAE	2018	250 000 drug-like molecules from ZINC; 108 000 molecules from QM9 data set under 9 heavy atoms	Autoencoder	4.2 M	https://github.com/aspuru-guzik-group/chemical_vae	[80]
SMILES-BERT	2019	18.7 million compounds sampled from ZINC	Encoder-only transformer	13 M	https://github.com/uta-smile/SMILES-BERT	[88]
ChemBERTa/ChemBERTa-v2	2020	250 000 drug-like molecules from ZINC	Encoder-only transformer	5–77 M	https://github.com/seyonechithrananda/bert-loves-chemistry	[99]
MolBERT	2020	1.27 million GuacaMol benchmark data set molecules	Encoder-only transformer	85 M	https://github.com/BenevolentAI/MolBERT	[100]
MegaMolBART	2021	1.45 billion ‘reactive’ molecules from ZINC under 500 Da and logP ≤ 5	Encoder-decoder transformer	45–230 M	https://github.com/NVIDIA/MegaMolBART	[101]
Molformer	2022	1.1 billion molecules from ZINC and PubChem	Encoder-only transformer	110 M	https://github.com/IBM/molformer	[102]
Chemformer/MolBART	2022	100 million molecules randomly sampled from ZINC under 500 Da and logP ≤ 5	Encoder-decoder transformer	45–230 M	https://github.com/MolecularAI/Chemformer	[90]
X-MOL	2022	1.1 billion ZINC database molecules	Encoder-decoder transformer	110 M	https://github.com/bm2-lab/X-MOL	[103]

One key feature of the transformer architecture is its ability to rapidly learn contextual information about input sequence data through a process called attention [82, 93]. This enables the weighting of an element in an input sequence in the context of its relative sequence position. Just like the meaning of a word is dependent on its position in a sentence and neighboring words, atoms affect molecular properties differently when they are in different positions and have different neighboring atoms. To inform these weighted contextual encodings, each input sequence element is augmented with a label based on its relative position. A typical transformer consists of attention layers combined with normalization and dense layers in ‘blocks’. Multiple encoder and decoder blocks are connected sequentially, resulting in billions of trainable parameters capable of considering both the identities of elements in a sequence as well as their relative positions.

The latent space occupied by these learned embeddings is highly organized. Indeed, impressive clustering of similar molecules at close points in latent space is observed as a result of unsupervised training [95]. While autoencoders and transformers can encode molecules into latent space through their encoder modules, they can also decode latent space vectors back into small molecules [96]. Hence, areas of latent space of interest (such as those containing known ligands for a given target) can be sampled, and decoded to produce novel small molecules in a generative manner [96, 97]. Early generative models faced the problem of generating molecules that were not accessible by chemical synthesis, which limited their adoption in practical applications [44, 98]. Nonetheless, generative models for small molecule design and drug discovery are an exciting and rapidly evolving field, enabling the systematic exploration of vast areas of chemical space.

### Transfer learning

Small data set sizes make finding optimal weights for large models difficult, which can fail to converge and reach high performance [87]. In such cases, simpler machine learning models like tree-based methods frequently perform better [104]. Pretrained embedders, as discussed above, can leverage much more training data than these end-to-end downstream models, resulting in embeddings that are broadly applicable but may lack the specific detail needed for specific tasks. On the other hand, the embeddings produced within an end-to-end model are most relevant to the task at hand but may fail to generalize across chemical space. Transfer learning is a recent development to make the best from this dichotomy [105, 106].

Transfer learning is a form of semi-supervised learning: it couples an unsupervised pretraining step with a subsequent supervised fine-tuning step [105]. Often, a given pretrained model is combined with a prediction head (often a linear layer taking the embedded representation as input and producing a predicted label as an output). This expanded model is then trained in a supervised manner on a smaller data set relevant to the desired downstream task [107]. For example, a transformer model could be trained on the entire ZINC database using masking, and then subsequently trained on a smaller data set to predict solubility, toxicity or other molecular properties using only the encoder module and an additional layer for property classification/regression. Pesciullesi *et al.* [108] successfully applied transfer learning to fine-tune a SMILES-based transformer to predict chemical reaction products of carbohydrates. Li and Fourches [109] leveraged transfer learning to achieve high performance on lipophilicity prediction, blood–brain barrier penetration and several other small molecule benchmarks. Jablonka *et al*. recently fine-tuned GPT-3 [110] on various molecular regression, classification and inverse design tasks, and showed promising results in the low-data regime [111].

The transfer learning approach has multiple advantages. First, much more data can be leveraged in the pretraining step because of its unsupervised nature, exposing the model to a much larger range of small molecules than would have been seen with traditional end-to-end training. Second, this large amount of initial data allows the embedder to converge on sensible weights during pretraining to produce molecular embeddings. In this way, during the fine-tuning step, most of the model has sensible initial weights and the loss can mainly be used to optimize the weights for the added prediction head, with little fine-tuning of the pretrained embedder. Since parameters are changed less based on the small, labeled data set, convergence to a high-performing combination of weights is easier [105, 112]. Although transfer learning involves pretraining a single model, which can then be reused and adapted to multiple downstream tasks, the pretraining of the model can be very expensive, complex, lengthy and energy intensive [113].

### Large language models

Recently, LLMs like GPT-4 [94, 114] have been trained on natural language text, such as English, and shown emergent capabilities across domains. Their ability to reason over unstructured text has great potential for extracting relevant information from chemical literature, where standardization is often lacking. Moreover, multimodal foundation models [115], which capture information not only from text but also images, could further improve the literature knowledge extraction. For chemical text generation, question answering and task solving, however, LLMs trained on immense textual corpora typically still lack basic chemical reasoning [114, 116]. For instance, molecular structures and IUPAC names are often hallucinated. While answers to chemical questions might sound convincing, they are often inaccurate or wrong and should always be verified. One attempt to overcome the weaknesses of LLMs is to give them access to external tools [117], such as calculators, a Python interpreter or a web search tool. For chemical purposes, Bran *et al.* [116] introduced ChemCrow, which augments GPT-4 with expert-designed tools for molecules, synthesis planning and execution, safety assessments and general applications. Given a user input, ChemCrow autonomously queries relevant expert-designed tools, and combines their responses, and solves challenging chemistry tasks, which were out of reach for GPT-4. With a different focus, Boiko *et al.* [118] show that LLMs can query the web, read documentation and write code for launching experiments on robotic platforms.

When used with responsibility, such LLM agents can lower the barrier for non-experts to access various powerful tools and information efficiently leading to a speed-up of research processes [116]. However, by modifying the LLM agents’ aims, there can be significant risks for dual use [119]. How to best put guardrails in place and mitigate those risks should be discussed with a wider community [120].

## CONCLUSIONS

A key goal for AI scientists and cheminformaticians in drug discovery is to reduce costs, time and failure rates. Despite the current high costs for drug discovery, low approval rates and reproducibility challenges [121–124], there are reasons for optimism. Biotech and pharma are expanding toward new modalities, such as proximity-induced neobiology, natural products, biologics and other macromolecules [125–129], and they are doing so using very powerful tools, such as DNA-encoded libraries, high-content imaging, multi-omics and AI-powered screens [130–132]. This creates needs and opportunities for novel molecular representations.

First, limitations in the size of available data sets constrain the ability to model many facets of drug discovery, particularly those involving the costliest experiments. While end-to-end deep learning models tend to overfit small data sets, the incorporation of prior knowledge can enhance the models’ generality. One such approach involves transfer learning: pretraining the models on large data sets to extract general features, which are subsequently fine-tuned for specific tasks. Despite significant advancements in pretraining models at the molecular level [88, 133], most current pretraining methods are limited to extracting features from the molecule itself but ignore the interactions between the molecule and other biological entities such as targets and diseases. Pretraining at the molecule network level remains an area of limited exploration. Indeed, in the field of drug discovery it is likely the level of high quality, unbiased input data is a major limitation rather than the sophistication of the available machine learning architectures. Future efforts in this space require, not just designing and evaluating increasingly complex and novel architectures, but also identifying and curating data to better inform current models [134].

Knowledge graphs (KGs) are multi-relational networks that store interlinked descriptions of different entities. Broad biomedical KGs have been well reported recently [135, 136], helping understand complex biological systems and pathologies. Integrating KGs information into molecular representation learning offers a powerful means of capturing the complex relationships between different entities and can aid in the representation of important structural and functional relationships between molecules. Recent work [137] has demonstrated the efficacy of incorporating KG pretraining strategies to address issues of small training sets in reaction yield prediction, achieving strong generalization abilities even with limited data. Additionally, prior knowledge can be derived from a variety of sources, including proteomics, metabolomics and phenotypic features in response to compound treatment such as pathway activity and modes of action [138]. Moreover, the combination of AI and physical simulations is increasingly fruitful thanks to the improved understanding of biological systems and the availability of supercomputers, GPUs, TPUs and other quasi-ASIC acceleration hardware. Although encoding molecular information into descriptors, fingerprints and deep learning models remains a challenge, the combination of different representations through multi-view techniques [139] can help overcome biases inherent to individual representations.

An alternative strategy to cope with the limitations of small data or even zero data is the combination of AI and physical simulations. Based on first principles, physical simulation provides valuable domain knowledge for the development of AI models [140]. Additionally, the prediction of AI can be effectively validated by simulation through supercomputers, leading to further adjustment and promotion of the model. We believe such a closed loop holds great potential for AI in drug discovery.

Second, there is a need for representations that are interpretable. Despite their expressive power in molecular representation learning across molecular size ranges, sequence- and graph-based deep learning methods lack interpretability. There are efforts to create fingerprints suitable for larger, more complex molecules [16, 141]. Compared with the learned representations that require a vast amount of data, bespoke descriptors are designed by experts, which make them easy to interpret and analyze. For small data sets, bespoke descriptors can often offer competitive performance, along with good interpretability and low computational cost, but they may also be biased toward the expert’s understanding of the problem. For example, Jiang *et al.* [104] demonstrated comparable performance to a GCN approach on several benchmark data sets using descriptor-based features combined with statistical and simple machine learning-based approaches. Interestingly, in recent work, XAI-FP [142] (explainable artificial intelligence-assisted fingerprints) encoded learned representations into fingerprints, showing that the combination of interpretable descriptors and learned representation can improve the quality of molecular representations.

Third, there is a need for molecular representations better suited to new therapeutic modalities. Beyond small-molecule drugs, larger therapeutics are gaining increasing relevance because of their potential to treat some conditions more safely or efficaciously. Their larger size and complexity allow them to address different targets, engage novel mechanisms or achieve improved delivery and specificity. Accurately predicting the properties of therapeutic classes such as nucleic acids, peptides and proteolysis-targeting chimeras (PROTACs) becomes necessary. To note some promising directions, Parisien *et al.* [143] constructed the MC-Fold and MC-Sym pipelines for inferring RNA secondary structure from raw sequences to guide the development of nucleic acid aptamers. Hansen *et al.* [129] created an algorithm to search for cell-penetrating peptides in proteins or random peptide sequences based on new chemical descriptors related to bioactive peptide design, providing new opportunities for developing peptide-based intracellular delivery carriers. PROTACs have been developed to accelerate the degradation of target cellular proteins the ubiquitin-proteasome system [144]. Because of their fuzzy structure–activity relationship and the frequent involvement of protein–protein interactions, the rational design of PROTACs poses significant challenges. Li *et al.* [145] introduced a deep neural network model to generate graph representations of ligands and their binding pockets, inputting them into a graph CNN for feature extraction, whereas the SMILES representation of the linker was fed into a bidirectional LSTM layer to generate features. The model achieved good prediction accuracy on the test set.

Lastly, advances in natural language processing have shown the extent to which leveraging unlabeled background information can lead to impressive performance in general language tasks, demonstrating the power of current models and architectures. Integrating all existing chemical knowledge into a model could similarly lead drug discovery to new heights. Unlike natural language, chemistry knowledge is fragmented across very different types of encodings, from chemical formulas to digital representations, mathematical equations and natural language, making its integration and harmonization difficult. Compared with text, high-quality chemical data are more scarce and costly to generate. Advances in automation and self-driving labs, in which artificial intelligence and active learning are used to guide experimental design, could enable higher throughput data generation leading to better models, more accurate predictions and more effective drug discovery with higher clinical translation and success rates.

**Key Points**

- Conventional molecular descriptors are easy to use and can be competitive for small data sets.
- Autoencoder and transformer representations based on strings or molecular graphs are highly popular.
- Transfer learning and physical simulations can help alleviate the limitations of small data sets.
- Broader knowledge integration including large-language models could enable novel applications.

## Data Availability

This study does not produce or analyze new data.
